# Sex differences in patients with repaired tetralogy of Fallot support a tailored approach for males and females: a cardiac magnetic resonance study

**DOI:** 10.1007/s10554-020-01900-x

**Published:** 2020-05-30

**Authors:** Quint A. J. Hagdorn, Niek E. G. Beurskens, Thomas M. Gorter, Graziëlla Eshuis, Hans L. Hillege, George K. Lui, Scott R. Ceresnak, Frandics P. Chan, Joost P. van Melle, Rolf M. F. Berger, Tineke P. Willems

**Affiliations:** 1 Center for Congenital Heart Diseases, Department of Pediatric Cardiology, University Medical Center Groningen, University of Groningen, Groningen, The Netherlands; 2 Heart Center, Department of Clinical and Experimental Cardiology, Amsterdam UMC, University of Amsterdam, Cardiovascular Sciences, Amsterdam, The Netherlands; 3 Center for Congenital Heart Diseases, Department of Cardiology, University Medical Center Groningen, University of Groningen, Groningen, The Netherlands; 4 Department of Epidemiology, University Medical Center Groningen, University of Groningen, Groningen, the Netherlands; 5 Departments of Medicine and Pediatrics, Divisions of Cardiovascular Medicine and Pediatric Cardiology, Stanford University School of Medicine, Palo Alto, CA USA; 6 Division of Pediatric Cardiology, Stanford University School of Medicine, Palo Alto, CA USA; 7 Department of Radiology, Stanford University Medical Center, Stanford University School of Medicine, Palo Alto, CA USA; 8 Department of Radiology, University Medical Center Groningen, University of Groningen, Groningen, The Netherlands

**Keywords:** Tetralogy of Fallot, Cardiac magnetic resonance, Sex, Sex differences, Pulmonary valve replacement

## Abstract

*Purpose* Substantial differences between sexes exist with respect to cardiovascular diseases, including congenital heart disease. Nevertheless, clinical decisions in the long-term follow-up of patients with repaired tetralogy of Fallot (rTOF) are currently based on unisex thresholds for cardiac magnetic resonance (CMR) measurements. This study aimed to assess whether sex differences exist in cardiac adaptation to hemodynamic loading conditions in patients with rTOF. *Methods and Results* This cross-sectional, two-center, combined pediatric and adult cohort included 320 rTOF patients (163 males, 51%) who underwent routine CMR. Despite similar age (median and interquartile range [m + IQR] 23.4 [15.2–34.4] years), surgical history, and hemodynamic loading, males with rTOF demonstrated higher biventricular CMR-derived volumes and masses, indexed for body surface area, compared to females (e.g. m + IQR right ventricular (RV) end-diastolic volume: males 123 [100–151] mL/m^2^, females 114 [94–131] mL/m^2^, P = 0.007). Sex-specific Z-scores of biventricular volumes and masses were similar for males and females. RV volumes and masses correlated with hemodynamic loading, but these relations did not differ between sexes. Biventricular ejection fraction (EF) appeared to be lower in male patients, compared to female patients (e.g. m + IQR RVEF: males 48 [43–54]%, females 52 [46–57]%, P < 0.001). *Conclusion* Indexed ventricular volumes and masses are higher in males with rTOF, compared to females, similar to the healthy population. RV hypertrophy and dilatation correlated to loading conditions similarly for both sexes. However, under comparable loading conditions, males demonstrated more severe functional impairment. These results indicate that sex-differences should no longer be ignored in treatment strategies, including timing of pulmonary valve replacement.

**Electronic supplementary material:**

The online version of this article (10.1007/s10554-020-01900-x) contains supplementary material, which is available to authorized users.

## Introduction

Tetralogy of Fallot (TOF) is one of the most prevalent congenital heart diseases, with excellent and mostly symptom-free survival into adulthood after surgical repair in the current era [1, 2]. However, patients remain at risk of progressive right ventricular (RV) dilatation and deterioration of cardiac function on the longer term [2, 3]. Timing of major clinical decisions, for example pulmonary valve replacement (PVR), is therefore key [4]. Cardiac magnetic resonance (CMR) derived measures of ventricular morphology and function form the cornerstone of decision making in the long-term follow-up of patients with repaired TOF (rTOF).

Currently, timing of PVR, for instance, is heavily based on CMR-derived RV volume thresholds, without distinction between sexes [5, 6]. However, it has been increasingly recognized in recent years that substantial differences exist between healthy men and women with respect to cardiac volumes and masses. In the healthy population, even after indexing for body surface area (BSA), males appear to have approximately 10% higher indexed volumes of both ventricles, and approximately 20% higher indexed mass of both ventricles, compared to women [7–9]. Ejection fraction (EF) did not differ between healthy men and women in these reports. Also in patients with congenital heart disease (CHD), relevant sex differences are increasingly recognized [10]. For example, females are more frequently symptomatic [11] and are more prone to develop pulmonary hypertension [12, 13], whereas the occurrence of arrhythmia and mortality is higher in males [14–16]. Further, similar to the healthy population, male rTOF patients appear to have higher biventricular volumes and mass (indexed for BSA) compared to female patients, despite equal hemodynamic burden and surgical history [17, 18].

The observation that biventricular volumes and mass differ substantially, both in the healthy population and the rTOF population, even when indexed for BSA, suggests that indication thresholds for interventions should be sex-specific [19]. However, the course of cardiac dimensions per sex in the rTOF population beyond the age of 20 years, relatively to healthy subjects, has not been described to date. Furthermore, it is unknown whether biventricular adaptation to abnormal loading conditions in rTOF might be different for male and female patients. The aim of the present study was to assess whether sex differences exist in cardiac adaptation to loading conditions in a combined American and Dutch, and combined pediatric and adult cohort of patients with rTOF.

## Methods

### Study population

Patients with rTOF, who underwent CMR imaging between January 2007 and May 2016 in Stanford University Medical Center (SUMC) or University Medical Center Groningen (UMCG) were retrospectively included, as reported previously [16]. Pulmonary atresia and absent pulmonary valve were excluded. Only patients between 8 and 60 years of age were included to adhere to the lowest age for which normal values are available [7], and since above 60 years of age, patient numbers were insufficient to make adequate comparisons. The need for individual informed consent for this study was waived by institutional Medical Ethical Review Boards of participating sites. This study has been performed in accordance with the ethical standards laid down in the 1964 Declaration of Helsinki and its later amendments.

### Patient characteristics, echo- and electrocardiography

Patient characteristics (i.e. age, sex, length, height) and medical history (i.e. occurrence of palliative shunt, date and type of TOF repair, any re-intervention) were extracted from records. The echocardiographic study and electrocardiogram closest to CMR, only if performed within 3 months from CMR and without relevant interventions in the time between echocardiography and CMR, were identified. Pulmonary valve (PV) peak gradient was extracted from echocardiographic report. The electrocardiogram was used to measure QRS duration.

### CMR analysis

CMR images were analyzed by three observers (T.M.G., N.E.G.B., or Q.A.J.H.) using QMass 7.6 (Medis, Leiden, The Netherlands) to measure biventricular volumes and mass. Good intra- and inter-observer variability analyses of these three observers have been reported previously [16]. Short-axis epi- and endocardial borders of both ventricles were traced manually in end-systole and end-diastole, in accordance with guidelines of the Society of Cardiovascular Magnetic Resonance. RV outflow tract was included in RV volume until the PV. Trabecular mass and papillary muscle mass were included into ventricular mass using MassK mode (semi-automatic threshold-based segmentation) [20]. Pulmonary flow (phase-contrast) was measured using QFlow 5.6 (Medis, Leiden, The Netherlands), after which pulmonary regurgitant fraction (PRF) was calculated. Post-processing background offset correction was performed, based on previous experience [21]. Volumes and masses were indexed for BSA using Haycock’s formula.

### Healthy reference cohorts

As reference cohort of healthy subjects from 8 to 20 years of age, a cohort of 49 girls and 49 boys, previously reported, was used [7]. In this report, subjects were grouped into 8–16, and 16–20 years of age. As reference cohort of healthy subjects beyond 20 years of age, a previously reported cohort of 60 men and 60 women was used, of which RV and LV data are published separately [8, 9]. In these studies, subjects were grouped into 10-year age groups. Patients in the present study cohort were grouped accordingly. These healthy reference cohorts were chosen since, in accordance with our method of CMR analysis, threshold-based analyses were used to exclude papillary muscles and trabecular structures from ventricular volumes, and to include in ventricular masses. Z-scores were calculated for every patient, using sex-specific and age-group specific normal values from the reference cohorts.

### Statistics

Data were presented as numbers and percentages or median and interquartile range (IQR). Continuous variables were compared between sexes using Mann–Whitney *U* test, categorical variables were using Chi-squared test. One-sample Wilcoxon signed rank test was performed to assess whether Z-scores were significantly different from zero. To assess whether PVR might have biased results, subgroup analyses of patients with PVR, and patients without PVR, were performed. Linear regression of CMR variables with echo PV peak gradient, PRF and age was performed. For linear regression, CMR variables and Z-scores of CMR variables that were not normally distributed were transformed until normal distribution was reached (defined as visually normal distribution on histogram and Q-Q plot, and a Kolmogorov–Smirnov P-value > 0.05). To assess whether associations with PV peak gradient are different between sexes, linear regression of CMR variables was performed with PV peak gradient, sex, and an interaction term of PV peak gradient multiplied by sex. When the P value of the interaction term was < 0.05, we rejected the null hypothesis that sexes have similar associations with PV peak gradient. Similar procedures were used to assess differences in associations with PRF, age, and age at repair between sexes. To allow clinical interpretation of the data, B-coefficients of the untransformed CMR data were displayed. Statistical significance was considered achieved at a P value < 0.05.

## Results

### Study population

A total of 320 patients, consisting of 163 (51%) male patients and 157 (49%) female patients, were included. Of these, 112 patients (35%) were under the age of 18 years. Study population characteristics are displayed in Table 1. Age at CMR, age at TOF-repair, surgical history, body mass index (BMI), PRF, PV peak gradient and cardiac index were similar between males and females. Older patients received their surgical repair at an older age compared to younger patients, reflecting the evolving surgical practice and timing over time, but no sex bias was observed in the timing of surgery (Supplemental Fig. 1). PRF ranged from 0 to 80%, and PV peak gradient ranged from 0 to 85 mmHg. QRS duration and BSA were significantly higher in male patients.

**Table 1:** Study population characteristics

	Male (n = 163)	Female (n = 157)	P value
*Patient characteristics*			
Age (years)	23.0 [15.7–33.5]	23.9 [10.1–52.8]	0.965
BMI (kg/m^2^)	23.8 [20.0–26.0]	22.2 [19.7–25.6]	0.084
BSA (m^2^)	1.9 [1.6–2.1]	1.6 [1.4–1.8]	** < 0.001**
*History*			
Age at repair (years)	1.3 [0.0–13.0]	1.4 [0.0–11.0]	0.877
Shunt palliation	32 (19.6%)	36 (22.9%)	0.471
Correction type			0.955
- No TAP	25 (15.3%)	25 (15.9%)	
- TAP	86 (52.8%)	88 (56.1%)	
- Conduit	5 (3.1%)	6 (3.8%)	
- Unknown	47 (28.8%)	38 (24.2%)	
PVR prior to CMR	32 (19.6%)	26 (16.6%)	0.476
Redo-PVR prior to CMR	3 (1.8%)	2 (1.3%)	0.683
Age at first PVR (years)	24.7 [16.5–37.6]	23.7 [14.6–36.6]	0.832
*Electrocardiography (n = 275)*			
QRS duration (ms)	148.5 [124.0–164.5]	138.0 [116.0–152.0]	**0.003**
*Cardiac magnetic resonance PC-flow*			
PRF (%)	30.0 [16.0–42.0]	28.8 [17.0–38.8]	0.509
Cardiac index (L/min/m^2^)	3.0 [2.5–3.6]	3.1 [2.6–3.7]	0.382
*Echocardiography (n = 241)*			
PV peak gradient (mmHg)	20.2 [12.4–33.3]	19.0 [13.1–29.6]	0.830

*BMI* body mass index, *BSA* body surface area, *TAP* trans-annular patch, *PVR* pulmonary valve replacement, *CMR* cardiac magnetic resonance, *PC* phase-contrast, *PRF* pulmonary regurgitant fraction, *PV* pulmonary valve. P values below 0.05 are displayed in bold.

### CMR variables compared to normal

Table 2 displays CMR variables, either indexed for BSA, or expressed as Z-score relative to healthy subjects. Regarding the RV, both male and female patients with rTOF demonstrate substantially higher indexed RV end-diastolic volume (RVEDVi), higher indexed RV mass (RVMi), and lower RV ejection fraction (RVEF), compared to healthy subjects. Regarding the LV, both male and female patients with rTOF demonstrate decreased LV ejection fraction (LVEF), and a mildly decreased indexed LV end-diastolic volume (LVEDVi) and indexed LV mass (LVMi), compared to healthy subjects.

**Table 2:** CMR variables by sex

	Indexed CMR variables		P value	Z-scores of CMR variables		P value
	Male	Female		Male	Female	
LVEF (%)	57.7 [52.0–62.6]	60.0 [54.9–65.0]	**0.013**	− 1.60 [− 2.80 to − 0.43]	− 0.87 [− 2.05 to 0.00]	**0.003**
LVEDVi (mL/m^2^)	80.6 [67.3–92.6]	73.1 [63.3–85.6]	**0.002**	− 0.44 [− 1.82 to 0.78]	− 0.68 [− 1.71 to 0.54]	0.726
LVESVi (mL/m^2^)	34.5 [25.2–42.2]	28.6 [24.2–35.6]	**0.001**	0.67 [− 0.65 to 2.13]	0.15 [− 0.68 to 1.54]	0.219
LVMi (g/m^2^)	57.5 [49.9–68.8]	47.3 [41.6–55.1]	**< 0.001**	− 0.84 [− 2.20 to 0.20]	− 1.12 [− 2.08 to to − 0.16]	0.462
RVEF (%)	47.7 [42.8–54.1]	52.3 [46.4–57.3]	**< 0.001**	− 2.65 [− 3.52 to − 1.46]	− 2.10 [− 3.00 to − 0.97]	**0.001**
RVEDVi (mL/m^2^)	122.5 [99.5–151.4]	114.4 [94–131.1]	**0.007**	3.00 [1.10 to 5.58]	3.72 [1.36 to 5.49]	0.339
RVESVi (mL/m^2^)	64.4 [48.1–79.7]	52.5 [41.4–66.8]	**< 0.001**	4.57 [2.31 to 6.74]	3.84 [2.30 to 6.35]	0.210
RVMi (g/m^2^)	44.4 [36.1–52.1]	34.3 [29.7–41.2]	**< 0.001**	2.27 [1.38 to 3.33]	2.10 [0.42 to 3.61]	0.340

*CMR* cardiac magnetic resonance, *LVEF LV* ejection fraction, *LVEDVi LV* indexed end-diastolic volume, *LVESVi LV* indexed end-systolic volume, *LVMi LV* indexed mass, *RVEF RV* ejection fraction, *RVEDVi RV* indexed end-diastolic volume, *RVESVi RV* indexed end-systolic volume, *RVMi RV* indexed mass

The displayed P-values are from male–female comparisons. The medians of all Z-scores are significantly different from zero (P < 0.001, not displayed). P values below 0.05 are displayed in bold.

### Sex differences in CMR variables

Indexed CMR variables all differed significantly between sexes (Table 2). Roughly, males appeared to have 10% higher indexed ventricular volumes, 20% higher indexed ventricular masses and lower EF of both ventricles. However, when normalized for sex and age (Z-score), only RVEF and LVEF were lower in males compared to females. To be able to compare with previous studies, subgroup analysis of patients within the age range of 8 to 20 years was performed [7, 17]. To assess whether sex differences are similarly present in young children, subgroup analysis of patients within the age range of 8 to 12 years was performed. Furthermore, to assess whether PVR might have influenced the described results, subgroup analyses of patients with and without PVR were performed. All these subgroup analyses demonstrate results that are similar to the complete study cohort (Supplemental Table 1).

### Association with RV pressure load, RV volume load, age and age at repair

**Fig. 1:**
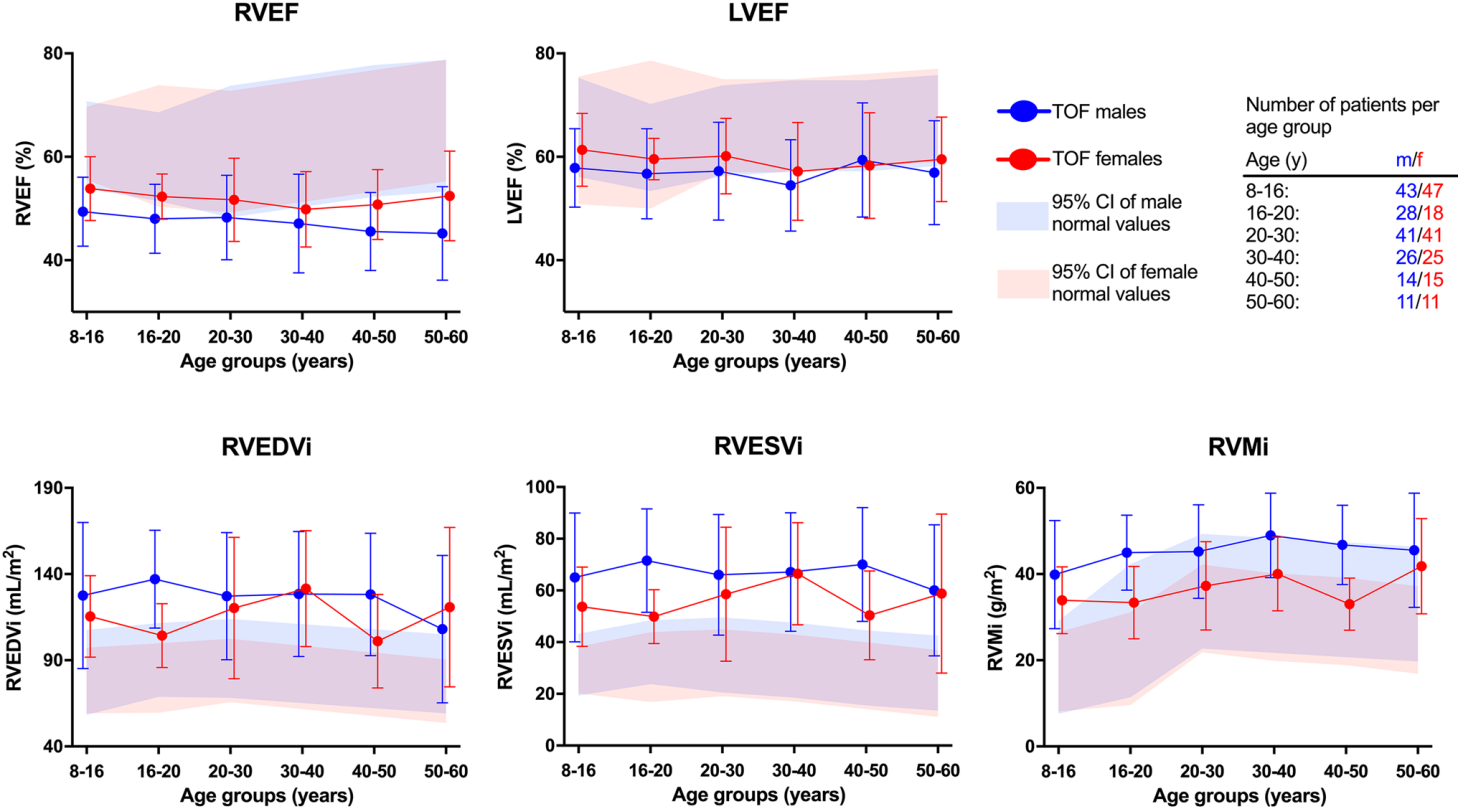
Sex-specific CMR variables in various age groups of patients with rTOF. Median and interquartile range of rTOF patients in blue (males) or red (females), superimposed on 95% confidence interval (1.96 SD) of healthy children and adolescents (8–20 years) [7] and adults (20–60 years) [8, 9] per age group. *RVEF RV* ejection fraction, *LVEF LV* ejection fraction, *RVEDVi RV* indexed end-diastolic volume, *RVESVi RV* indexed end-systolic volume, *RVMi RV* indexed mass

Associations with PV peak gradient, PRF, age, and age of repair are displayed in Table 3. All RV, but no LV variables correlated with RV pressure load, represented by PV peak gradient: RVEF and RVMi were higher at higher RV pressure load, whereas RV volumes were lower at higher RV pressure load. These associations did not differ significantly between sexes. Furthermore, LV variables correlated with RV volume load, represented by PRF: LVEF and LVMi were lower at greater RV volume load, while RV volumes and RVMi were higher at greater RV volume load. Both RV and LV mass were higher at higher age, whereas RVEF was lower at higher age. Neither of these associations differed significantly between sexes. None of the CMR variables correlated with age at repair. Linear regression analyses were additionally performed with Z-score values, with similar results, as displayed in Supplemental Table 2. The only relevant difference was that LVEF Z-score, but not LVEF, was lower at higher age (B-coefficient − 0.032, P value < 0.001, R^2^ 0.045), indicating that older patients with repaired TOF demonstrate more impairment of biventricular systolic function compared to younger patients. No associations between CMR variables and age at TOF correction were demonstrated (not displayed). A graphical depiction of indexed CMR variables in different age groups, superimposed on normal values, is provided in Fig. 1 and Supplemental Fig. 2. Median Z-scores of different age groups are displayed in Supplemental Fig. 3.

**Table 3:** Linear regression with PV peak gradient, PRF, age and age at repair

	B-coefficient	P value	R^2^	Interaction with sex (P value)
PV peak gradient				
LVEF (%)	0.052	0.169	0.008	0.592
LVEDVi (mL/m^2^)	− 0.049	0.496	0.002	0.943
LVESVi (mL/m^2^)	− 0.081	0.148	0.009	0.890
LVMi (g/m^2^)	0.054	0.421	0.003	0.772
RVEF (%)	0.070	**0.026**	0.021	0.971
RVEDVi (mL/m^2^)	− 0.365	**0.019**	0.023	0.820
RVESVi (mL/m^2^)	− 0.262	**0.003**	0.036	0.859
RVMi (g/m^2^)	0.089	**0.028**	0.020	0.722
PRF				
LVEF (%)	− 0.072	**0.021**	0.017	0.290
LVEDVi (mL/m^2^)	− 0.093	0.115	0.008	0.728
LVESVi (mL/m^2^)	0.009	0.938	< 0.001	0.620
LVMi (g/m^2^)	− 0.160	**0.005**	0.026	0.896
RVEF (%)	− 0.004	0.882	< 0.001	0.842
RVEDVi (mL/m^2^)	1.219	**< 0.001**	0.344	0.505
RVESVi (mL/m^2^)	0.636	**< 0.001**	0.225	0.488
RVMi (g/m^2^)	0.204	**< 0.001**	0.095	0.685
Age				
LVEF (%)	− 0.046	0.300	0.003	0.517
LVEDVi (mL/m^2^)	− 0.025	0.747	< 0.001	0.197
LVESVi (mL/m^2^)	0.022	0.735	0.000	0.277
LVMi (g/m^2^)	0.279	**< 0.001**	0.058	0.224
RVEF (%)	− 0.080	**0.016**	0.018	0.794
RVEDVi (mL/m^2^)	− 0.043	0.442	0.002	0.442
RVESVi (mL/m^2^)	0.079	0.752	< 0.001	0.566
RVMi (g/m^2^)	0.151	**0.001**	0.033	0.742
Age at repair				
LVEF (%)	− 0.135	0.170	0.006	0.162
LVEDVi (mL/m^2^)	− 0.383	0.052	0.013	0.763
LVESVi (mL/m^2^)	− 0.062	0.656	0.001	0.405
LVMi (g/m^2^)	− 0.136	0.052	0.013	0.062
RVEF (%)	− 0.126	0.140	0.007	0.535
RVEDVi (mL/m^2^)	− 0.363	0.148	0.007	0.437
RVESVi (mL/m^2^)	− 0.009	0.614	0.001	0.413
RVMi (g/m^2^)	0.182	0.135	0.008	0.211

*PV* pulmonary valve, *PRF* pulmonary regurgitant fraction, *LVEF LV* ejection fraction, *LVEDVi LV* indexed end-diastolic volume, *LVESVi LV* indexed end-systolic volume, *LVMi LV* indexed mass, *RVEF RV* ejection fraction, *RVEDVi RV* indexed end-diastolic volume, *RVESVi RV* indexed end-systolic volume, *RVMi RV* indexed mass. P values below 0.05 are displayed in bold.

## Discussion

The present study in a comprehensive cohort of patients with rTOF, ranging from 8 to 60 years of age, identified substantial differences in indexed CMR-derived measures of cardiac size and function between males and females. Whilst clinical characteristics, such as age, surgical history, and loading conditions of the RV did not differ between sexes, males appeared to have longer QRS duration and about 10% higher left and right ventricular volumes and 20% higher right and left ventricular masses, even after indexing for BSA, when compared to females. These differences are similar to differences in the healthy population, where males also have larger and heavier hearts [7–9]. After normalizing for sex, by creating sex-specific Z-scores according to healthy reference values, no differences in volumes and masses between males and females with rTOF remained. This indicates that male and female patients with rTOF demonstrate a similar deviation from healthy subjects, and thus demonstrate similar cardiac remodeling in response to comparable loading conditions.

Surgical history and hemodynamic burden were compared between sexes to assess whether they might have biased results. At first, hemodynamic burden at the time of CMR was demonstrated to be equal between sexes. Furthermore, no differences in timing of repair surgery, type of repair surgery and frequency of the need for shunt palliation were observed between sexes (Table 1 and Supplemental Fig. 1). This demonstrates that there is no indication that disease severity or treatment strategies differed between sexes. Also after initial surgical repair, no difference in the timing or frequency of PVR were observed as the percentage of patients with PVR and the age at first PVR was similar between men and women. Finally, the observed ratio male:female did not differ between both the subgroup of patients without PVR (131 males, 131 females), and the subgroup of patients who received PVR (32 males, 26 females, Supplemental Table 1). Together, these findings suggest that history and disease severity did not form a confounding factor in this study.

The degree of RV dilatation (RVEDVi) and RV hypertrophy (RVMi) correlated with volume- and pressure load respectively. RV volumes were smaller at higher RV pressure load, and larger with increasing RV volume load, indicative of concentric and eccentric remodeling, respectively. RV mass was higher with both increasing RV pressure and volume load. The association of increasing pressure load with hypertrophy, decreasing RV volumes and increasing RVEF probably reflects compensated concentric hypertrophic remodeling in the minority of patients with significantly increased PV gradient. Interestingly, LVEF and LV mass were lower with increasing RV volume load. However, none of these associations between cardiac measures and ventricular loading conditions or age differed significantly between sexes. These findings suggest that male and female rTOF patients respond similarly to abnormal loading conditions of the RV and increasing age with respect to ventricular volumes and mass.

With respect to ventricular function, both RVEF and LVEF were decreased in patients with rTOF, compared to healthy reference values. This observation is in line with previous reports [3, 17]. After normalizing for sex-specific healthy reference values (by means of Z-scores), this decrease in biventricular EF was larger in males compared to females with rTOF. This is in contrast to the healthy population, where LVEF and RVEF do not differ between men and women [7–9]. Since biventricular EF is already lower in the subgroup of rTOF patients within 8–12 years of age, it seems unlikely that comorbidities, such as atherosclerotic disease, can be held accountable for the lower EF in male patients. The unfavorable functional adaptation of male patients in comparison to females could have consequences for outcome. For example, our group and others have demonstrated previously that male sex is a risk factors for arrhythmia in rTOF [12, 14–16]. While remaining speculative, this could indicate that the unfavorable functional adaptation in males contributes to an increased risk of arrhythmia. Taken together, functional adaptation of the RV is impaired in male rTOF patients, when compared with female patients with similar age, surgical history, RV loading conditions and similar degrees of RV dilatation and RV hypertrophy.

These findings seem to be in striking contrast with those of Sarikouch et al. who also found lower RVEF in males with rTOF compared to females, but after normalization for sex, reported a lower Z-score of RVEF in females compared to males [22]. This is remarkable and difficult to understand, since the cohort of this study, and that of Sarikouch et al. show very similar mean RVEF values, and use the same healthy reference cohort for Z-score calculation. Review of these published reports shows that in the healthy reference cohorts, there were no significant differences in RVEF between sexes, whereas in the rTOF population of the Sarikouch-cohort, RVEF was significantly lower in male patients compared to female patients (48.9% ± 6.7 in men, 53.4% ± 5.8 in women, P < 0.001). Then, it is hard to explain that the RVEF Z-score in men suddenly is higher than that in women. Thus, the lower RVEF Z-scores in men, described in the present study, are more plausible to adequately depict a more pronounced decrease of RVEF in male patients compared to female patients under similar conditions.

Sex hormones may play a role in these observed differences in biventricular systolic function between men and women with rTOF. In cardiovascular disease-free subjects, a large study has demonstrated that higher levels of estradiol were associated with higher RVEF [23, 24]. Furthermore, animal studies suggest that estrogen can preserve both RV and LV contractility [25–27]. This protective characteristic of female sex hormones might also be observed in pulmonary arterial hypertension, where female patients demonstrate improvement of RVEF after treatment, whereas males demonstrate deterioration, despite comparable baseline disease severity [28]. However, the observed differences between males and females were already present in patients younger than 12 years of age. Most patients in this age group are pre-pubertal, and although some patients may have reached puberty prior to the age of 12, the amount of time exposed to post-pubertal levels of sex hormones is little. This suggests that, besides possible hormonal factors, other mechanisms additionally play a role in the differences between male and female patients. Taken together, evidence is increasing that female sex, or female sex hormones, beneficially influences cardiac function.

The commonly used absolute unisex criteria for RV volume as indication for PVR may erroneously cause female patients to reach operation indications later, at a more advanced stage of dilatation, compared to males. Furthermore, male patients with rTOF and dilated RV might need PVR earlier than female patients with similar dilatation, due to the occurrence of more or earlier deterioration of RV function. With this in mind, one could expect that men would receive PVR at a younger age, and more often, compared to female patients. In the current study, however, men and women demonstrated similar rates of PVR, and men were not younger at the time of first PVR. Speculatively, this might be due to women reaching other indications for PVR earlier than men. For example, female patients with CHD have lower exercise capacity, and are more frequently symptomatic, compared to male patients [11, 22]. This apparent discrepancy between this unfavorable clinical state in women with rTOF, whereas they have less impaired ventricular function, compared to men, requires further study. Also, it remains unknown if referral bias, or other sex biases in the physician’s approach towards patients, plays any role in this matter.

Moreover, one might expect that, if women reach operation thresholds at a relatively more advanced stage of RV dilatation compared to men, outcome after PVR would be less favorable in women. However, studies addressing outcomes after PVR are flawed by sex differences, since these studies use unisex CMR-derived criteria to assess RV normalization [29, 30]. Using such unisex criteria will result in women reaching the RV normalization definition earlier and thus presumably more frequent. Therefore, the previously reported finding that female sex is a favorable predictor for RV normalization after PVR in rTOF should be interpreted with suspicion [29].

The results of the current study have relevant consequences for clinical practice. The observed sex differences in the rTOF population are currently not taken into account in guidelines addressing the cutoff points for interventions, such as PVR, in patients with rTOF and residual lesions [5, 6]. The current findings implicate that diagnostic and treatment recommendations should be sex-specific, and both confirm and underscore previous suggestions in this direction [17–19, 31]. The present study demonstrates that, as long as RV dimensions and function are recommended and widely used as indication for PVR, sex-differences should no longer be ignored in treatment strategies. Future studies are needed to evaluate whether sex-specific treatment strategies can improve clinical outcomes.

### Limitations

This is a retrospective study, including patients over a 10-year period. A limitation of any cross-sectional study, relating measures of disease severity to age in the rTOF population, is that older patients have had their surgical repair and neonatal treatment in a different era and at different pre-repair conditions. These factors may confound the observed sex-related differences and could raise the question whether the observed differences in EF were caused by pre-repair differences or differential functional adaptation to abnormal loading. However, we found no differences between sexes with regards to patient characteristics, surgical history, age at repair and age at first PVR. Furthermore, only patients who underwent a CMR were included, which may have introduced a selection bias. However, as current guidelines prescribe CMR imaging as the modality of choice for assessment of RV volume and function, CMR imaging is performed routinely, limiting such biases [5]. Unfortunately, we did not have ethnicity nor history of pregnancy available. However, the present study describes lower RV volumes in female patients, compared to male patients, whereas pregnancy is known to increase RV volumes [32]. Therefore, it seems unlikely that the described differences between sexes result from pregnancy. However, the apparent increase in RVEDVi in females between the ages of 20 and 40 years may have been driven by an effect of pregnancy. This hypothesis can unfortunately not confirmed in the present study. Differences in all CMR variables are nevertheless present in all age groups, including the subgroup analysis of patients between 8 and 12 years of age. Also, despite that the current study included a relatively large and intercontinental cohort, numbers per sex in each age group are modest, limiting statistical power. At last, comparability with studies that calculate standard deviation scores, instead of Z-scores as calculated in the present study, may be limited due to different statistical approaches. However, it is unlikely that either method would cause systematic error, and therefore, it seems unlikely that this methodological discrepancy significantly influenced results.

## Conclusion

As in the normal population, ventricular volumes and masses in patients with rTOF, indexed for BSA, are substantially higher in males, compared to females, suggesting the same pattern of morphological adaptation to abnormal loading. However, under comparable loading conditions, biventricular systolic function was lower in male patients, compared to female patients. These results suggest that research and treatment recommendations need more focus on sex differences, including the use of sex-specific thresholds or Z-scores for timing of pulmonary valve replacement.

## Electronic supplementary material

Below is the link to the electronic supplementary material.

Supplementary file1 (DOCX 526 kb)
