# Supplementary material for: Sex differences in patients with repaired tetralogy of Fallot support a tailored approach for males and females: a cardiac magnetic resonance study
Source: Int J Cardiovasc Imaging. 2020 May 30;36(10):1997–2005. doi: 10.1007/s10554-020-01900-x (PMC7497497; doi:10.1007/s10554-020-01900-x)
Supplement: Supplementary file 1 — Supplementary file1 (DOCX 526 kb) [file 10554_2020_1900_MOESM1_ESM.docx]

**Supplemental material**


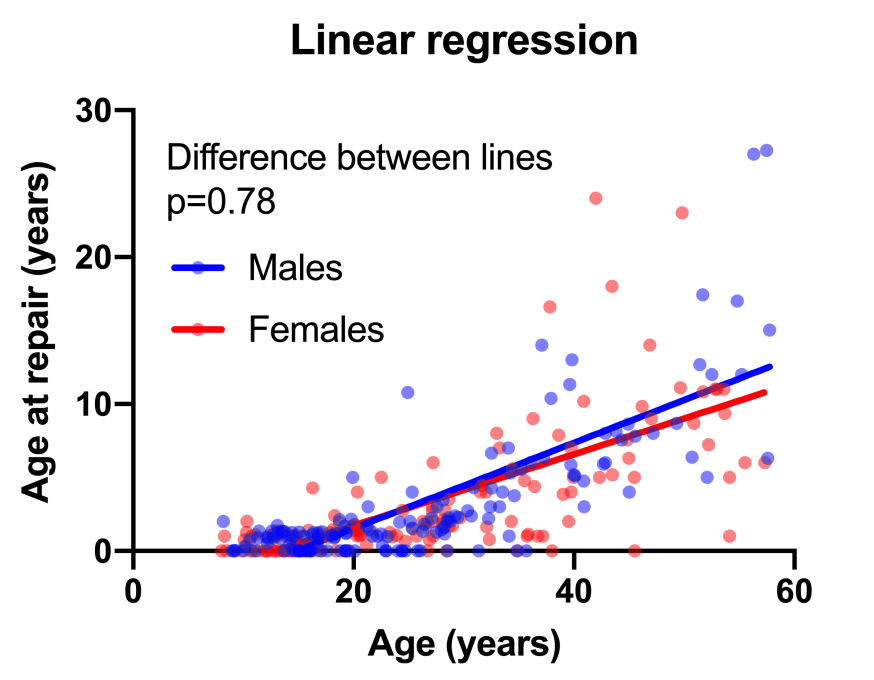


Supplemental figure 1: Linear regression of age and age at repair.


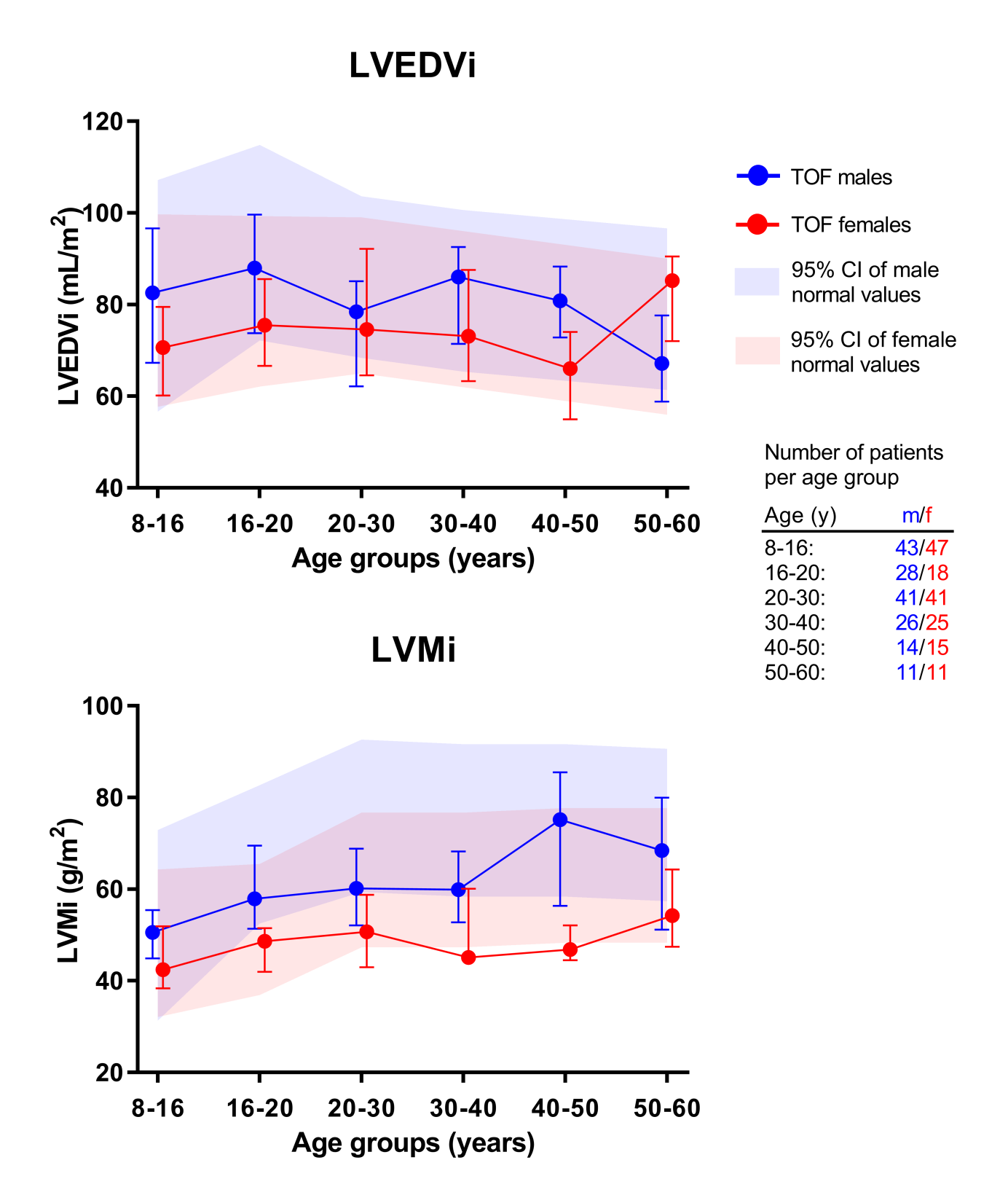
Supplemental figure 2: Sex-specific CMR variables in various age groups of patients with rTOF.

Median and interquartile range of rTOF patients in blue (males) or red (females), superimposed on 95% confidence interval (1.96 SD) of healthy subjects [7-9] per age group. LVEDVi LV indexed end-diastolic volume, LVMi LV indexed mass.


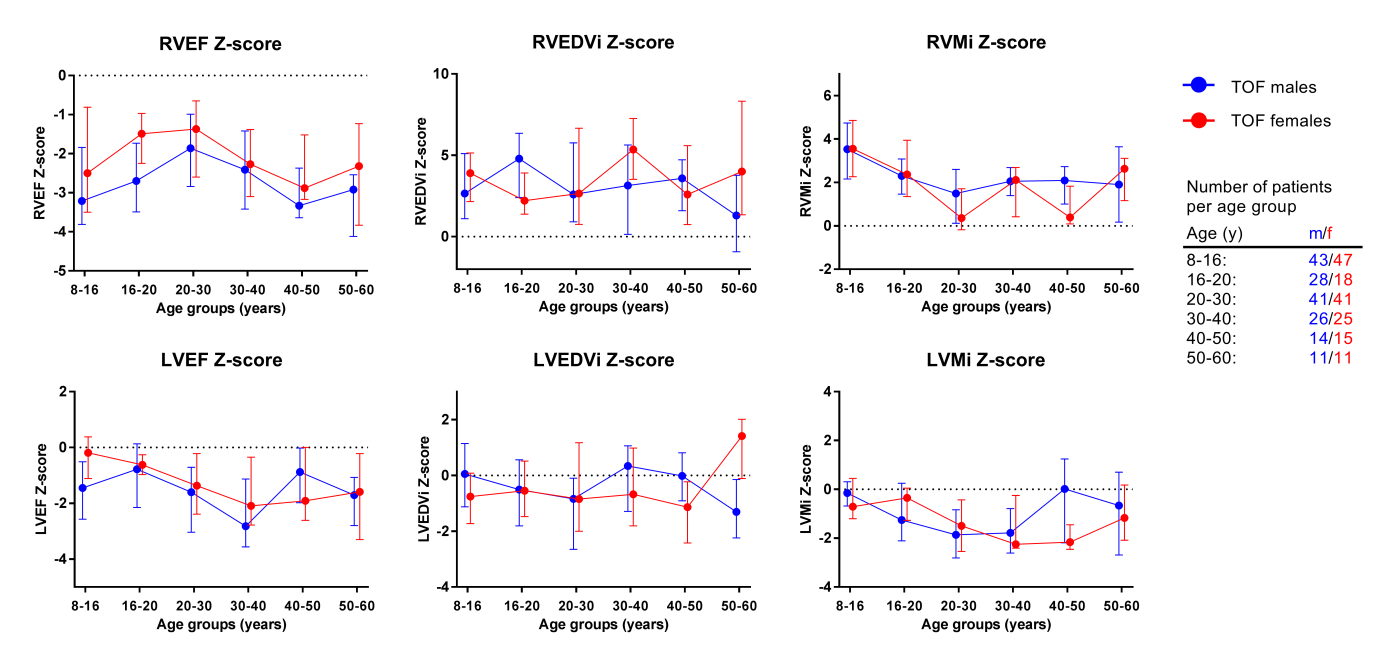


Supplemental figure 3: Sex-specific Z-scores of CMR variables in various age groups of patients with rTOF.

Median and interquartile range of rTOF patients in blue (males) or red (females) per age group. RVEF RV ejection fraction, RVEDVi RV indexed end-diastolic volume, RVMi RV indexed mass, LVEF LV ejection fraction, LVEDVi LV indexed end-diastolic volume, LVMi LV indexed mass.

| *Supplemental table 1a: CMR variables by sex, within an age range of 8 – 20 years* | | | | | | |
| --- | --- | --- | --- | --- | --- | --- |
|  | Indexed CMR variables | |  | Z-scores of CMR variables | |  |
|  | Male (n=71) | Female (n=65) | P value | Male | Female | P value |
| LVEF (%) | 58.6 [53.1 ‒ 62.6] | 61.1 [57.2 ‒ 64.9] | **0.025** | -1.12 [-2.33 ‒ -0.39] | -0.35 [-0.97 ‒ 0.27] | **<0.001** |
| LVEDVi (mL/m2) | 85.7 [69.8 ‒ 98.6] | 71.4 [63.0 ‒ 80.6] | **<0.001** | -0.14 [-1.57 ‒ 0.83] | -0.72 [-1.53 ‒ 0.08] | 0.138 |
| LVESVi (mL/m2) | 35.2 [25.8 ‒ 44.3] | 27.9 [24.3 ‒ 32.3] | **<0.001** | 0.22 [-0.59 ‒ 1.94] | -0.15 [-0.69 ‒ 0.46] | **0.021** |
| LVMi (g/m2) | 53.8 [46.4 ‒ 61.5] | 44.4 [39.7 ‒ 51.5] | **<0.001** | -0.34 [-1.18 ‒ 0.31] | -0.54 [-1.20 ‒ 0.33] | 0.573 |
| RVEF (%) | 48.5 [45.2 ‒ 54.4] | 53.6 [49.0 ‒ 57.7] | **<0.001** | -3.19 [-3.65 ‒ -1.84] | -2.13 [-3.02 ‒ -0.9] | **0.005** |
| RVEDVi (mL/m2) | 127.9 [101.6 ‒ 151.4] | 114.4 [97.3 ‒ 125.8] | **0.002** | 3.46 [1.48 ‒ 5.62] | 3.70 [1.77 ‒ 4.66] | 0.558 |
| RVESVi (mL/m2) | 66.5 [48.1 ‒ 77.9] | 51.7 [41.7 ‒ 61.3] | **<0.001** | 5.40 [2.75 ‒ 7.36] | 4.18 [2.37 ‒ 6.62] | 0.168 |
| RVMi (g/m2) | 40.7 [33.8 ‒ 48.5] | 34.0 [27.9 ‒ 40.2] | **<0.001** | 3.02 [2.00 ‒ 4.22] | 3.04 [2.10 ‒ 4.29] | 0.674 |

| *Supplemental table 1a: CMR variables by sex, within an age range of 8 – 12 years* | | | | | | |
| --- | --- | --- | --- | --- | --- | --- |
|  | Indexed CMR variables | |  | Z-scores of CMR variables | |  |
|  | Male (n=71) | Female (n=65) | P value | Male | Female | P value |
| LVEF (%) | 60.7 [53.0 ‒ 61.0] | 65.6 [61.7 ‒ 70.1] | **0.050** | -1.02 [-2.59 ‒ -0.96] | 0.38 [-0.24 ‒ 1.10] | **0.035** |
| LVEDVi (mL/m2) | 68.6 [57.8 ‒ 88.2] | 76.9 [62.7 ‒ 79.0] | **<0.001** | -1.03 [-1.87 ‒ 0.48] | -0.17 [-1.50 ‒ 0.03] | 0.670 |
| LVESVi (mL/m2) | 26.4 [23.8 ‒ 38.7] | 25.4 [18.7 ‒ 30.3] | **0.002** | -0.26 [-0.65 ‒ 1.56] | -0.57 [-1.67 ‒ 0.23] | 0.408 |
| LVMi (g/m2) | 45.8 [43.73 ‒ 53.6] | 41.8 [38.9 ‒ 44.4] | **0.002** | -0.59 [-0.79 ‒ 0.14] | -0.78 [-1.13 ‒ -0.47] | 0.590 |
| RVEF (%) | 47.3 [45.4 ‒ 50.9] | 55.4 [50.6 ‒ 59.5] | **<0.001** | -3.50 [-3.93 ‒ -2.65] | -2.00 [-3.33 ‒-0.86 ] | **0.004** |
| RVEDVi (mL/m2) | 104.5 [93.6 ‒ 131.9] | 118.9 [105.8 ‒ 128.1] | **0.002** | 1.72 [0.85 ‒ 3.89] | 4.19 [2.84 ‒ 5.14] | 0.719 |
| RVESVi (mL/m2) | 56.4 [45.2 ‒ 70.0] | 56.4 [42.5 ‒ 62.6] | **<0.001** | 4.11 [2.27 ‒ 6.34] | 5.90 [2.89 ‒ 7.26] | 0.098 |
| RVMi (g/m2) | 33.7 [29.7 ‒ 40.3] | 34.1 [28.1 ‒ 37.5] | **<0.001** | 2.80 [2.07 ‒ 4.00] | 3.58 [2.3 ‒ 4.29] | 0.181 |

| *Supplemental table 1c: CMR variables by sex, only patients without PVR* | | | | | | |
| --- | --- | --- | --- | --- | --- | --- |
|  | Indexed CMR variables | |  | Z-scores of CMR variables | |  |
|  | Male (n=131) | Female (n=131) | P value | Male | Female | P value |
| LVEF (%) | 57.0 [52.0 ‒ 62.6] | 59.6 [54.0 ‒ 65.0] | **0.026** | -1.78 [-2.89 ‒ -0.43] | -0.81 [-2.09 ‒ 0.00] | **0.004** |
| LVEDVi (mL/m2) | 79.1 [68.9 ‒ 92.5] | 73.3 [63.0 ‒ 85.8] | **0.016** | -0.46 [-1.76 ‒ 0.77] | -0.69 [-1.72 ‒ 0.67] | 0.959 |
| LVESVi (mL/m2) | 34.1 [25.5 ‒ 41.1] | 28.7 [24.2 ‒ 37.0] | **0.007** | 0.67 [-0.64 ‒ 1.96] | 0.21 [-0.66 ‒ 1.80] | 0.375 |
| LVMi (g/m2) | 55.4 [48.0 ‒ 65.1] | 46.2 [40.5 ‒ 53.6] | **<0.001** | -1.01 [-2.44 ‒ 0.03] | -1.17 [-2.19 ‒ -0.21] | 0.862 |
| RVEF (%) | 48.6 [44.3 ‒ 54.7] | 52.8 [46.5 ‒ 57.7] | **<0.001** | -2.46 [-3.49 ‒ -1.32] | -1.95 [-3.02 ‒ -0.86] | **0.009** |
| RVEDVi (mL/m2) | 128.0 [100.5 ‒ 158.5] | 116.5 [95.4 ‒ 137.5] | **0.024** | 3.45 [1.16 ‒ 6.18] | 3.92 [1.54 ‒ 6.10] | 0.268 |
| RVESVi (mL/m2) | 66.1 [47.7 ‒ 83.9] | 55.2 [41.4 ‒ 67.7] | **0.001** | 4.60 [2.14 ‒ 7.23] | 4.16 [2.30 ‒ 6.73] | 0.463 |
| RVMi (g/m2) | 44.2 [35.6 ‒ 52.3] | 35.0 [29.7 ‒ 42.0] | **<0.001** | 2.46 [1.41 ‒ 3.39] | 2.26 [0.53 ‒ 3.92] | 0.695 |

| *Supplemental table 1d: CMR variables by sex, only patients with PVR* | | | | | | |
| --- | --- | --- | --- | --- | --- | --- |
|  | Indexed CMR variables | |  | Z-scores of CMR variables | |  |
|  | Male (n=32) | Female (n=26) | P value | Male | Female | P value |
| LVEF (%) | 59.8 [56.7 ‒ 62.8] | 60.5 [57.3 ‒ 65.1] | 0.274 | -1.04 [-1.8 ‒ -0.43] | -1.05 [-1.78 ‒ 0.06] | 0.472 |
| LVEDVi (mL/m2) | 87.7 [63.8 ‒ 98] | 72.0 [65.8 ‒ 78.5] | 0.056 | -0.24 [-2.02 ‒ 1.04] | -0.59 [-1.71 ‒ -0.02] | 0.390 |
| LVESVi (mL/m2) | 35.5 [23.5 ‒ 44.4] | 28.2 [23.5 ‒ 33.0] | **0.038** | 0.59 [-0.78 ‒ 2.41] | 0.02 [-0.79 ‒ 1.12] | 0.302 |
| LVMi (g/m2) | 67.8 [56.9 ‒ 79.1] | 51.3 [46.3 ‒ 60.1] | **<0.001** | -0.25 [-1.74 ‒ 0.88] | -1.03 [-1.87 ‒ 0.06] | 0.205 |
| RVEF (%) | 44.7 [39.5 ‒ 47.6] | 49.1 [44.1 ‒ 54.1] | **0.008** | -3.30 [-4.01 ‒ -2.35] | -2.48 [-3.00 ‒ -1.78] | **0.038** |
| RVEDVi (mL/m2) | 115.1 [91 ‒ 128.6] | 97.0 [80.2 ‒ 108.9] | **0.025** | 2.31 [0.24 ‒ 3.44] | 1.74 [-0.40 ‒ 2.65] | 0.364 |
| RVESVi (mL/m2) | 63.5 [53.7 ‒ 73.1] | 48.6 [41.1 ‒ 55.4] | **0.001** | 4.41 [2.72 ‒ 5.68] | 2.75 [1.38 ‒ 4.89] | 0.070 |
| RVMi (g/m2) | 45.4 [38.7 ‒ 50.2] | 32.4 [28.4 ‒ 38.4] | **<0.001** | 1.88 [1.01 ‒ 3.06] | 1.17 [0.08 ‒ 1.99] | 0.065 |

Supplemental table 1: CMR cardiac magnetic resonance, LVEF LV ejection fraction, LVEDVi LV indexed end-diastolic volume, LVESVi LV indexed end-systolic volume, LVMi LV indexed mass, RVEF RV ejection fraction, RVEDVi RV indexed end-diastolic volume, RVESVi RV indexed end-systolic volume, RVMi RV indexed mass.

| **PV peak gradient** | B-coefficient | p value | R^2^ | Interaction with sex (P value) |
| --- | --- | --- | --- | --- |
| LVEF Z-score | 0.013 | 0.109 | 0.011 | 0.594 |
| LVEDVi Z-score | -0.004 | 0.573 | 0.001 | 0.972 |
| LVESVi Z-score | -0.015 | 0.101 | 0.011 | 0.990 |
| LVMi Z-score | 0.007 | 0.239 | 0.006 | 0.414 |
| RVEF Z-score | 0.011 | 0.060 | 0.015 | 0.339 |
| RVEDVi Z-score | -0.035 | **0.012** | 0.026 | 0.808 |
| RVESVi Z-score | -0.038 | **0.003** | 0.037 | 0.471 |
| RVMi Z-score | 0.019 | **0.011** | 0.022 | 0.117 |
|  |  |  |  |  |
| **PRF** | B-coefficient | P value | R^2^ | Interaction with sex (P value) |
| LVEF Z-score | -0.015 | **0.024** | 0.017 | 0.252 |
| LVEDVi Z-score | -0.010 | 0.085 | 0.010 | 0.499 |
| LVESVi Z-score | 0.001 | 0.995 | <0.001 | 0.742 |
| LVMi Z-score | -0.021 | **<0.001** | 0.048 | 0.335 |
| RVEF Z-score | -0.002 | 0.733 | <0.001 | 0.516 |
| RVEDVi Z-score | 0.111 | **<0.001** | 0.337 | 0.885 |
| RVESVi Z-score | 0.095 | **<0.001** | 0.225 | 0.950 |
| RVMi Z-score | 0.034 | **<0.001** | 0.092 | 0.507 |
|  |  |  |  |  |
| **Age** | B-coefficient | P value | R^2^ | Interaction with sex (P value) |
| LVEF Z-score | -0.032 | **<0.001** | 0.045 | 0.131 |
| LVEDVi Z-score | 0.008 | 0.298 | 0.003 | 0.276 |
| LVESVi Z-score | 0.029 | **0.004** | 0.026 | 0.194 |
| LVMi Z-score | -0.026 | **<0.001** | 0.042 | 0.406 |
| RVEF Z-score | -0.001 | 0.853 | <0.001 | 0.793 |
| RVEDVi Z-score | 0.008 | 0.939 | <0.001 | 0.274 |
| RVESVi Z-score | -0.009 | 0.497 | 0.001 | 0.830 |
| RVMi Z-score | -0.043 | **<0.001** | 0.080 | 0.936 |
|  |  |  |  |  |
| **Age at repair** | B-coefficient | P value | R^2^ | Interaction with sex (P value) |
| LVEF Z-score | -0.065 | **0.002** | 0.034 | 0.093 |
| LVEDVi Z-score | -0.016 | 0.428 | 0.002 | 0.965 |
| LVESVi Z-score | 0.034 | 0.142 | 0.007 | 0.420 |
| LVMi Z-score | -0.030 | 0.087 | 0.010 | 0.430 |
| RVEF Z-score | -0.015 | 0.367 | 0.003 | 0.854 |
| RVEDVi Z-score | -0.012 | 0.470 | 0.002 | 0.570 |
| RVESVi Z-score | -0.016 | 0.596 | 0.001 | 0.443 |
| RVMi Z-score | -0.052 | **0.019** | 0.019 | 0.373 |

Supplemental table 2: Linear regression of Z-scores with PV peak gradient, PRF, age and age at repair. PV pulmonary valve, PRF pulmonary regurgitant fraction, LVEF LV ejection fraction, LVEDVi LV indexed end-diastolic volume, LVESVi LV indexed end-systolic volume, LVMi LV indexed mass, RVEF RV ejection fraction, RVEDVi RV indexed end-diastolic volume, RVESVi RV indexed end-systolic volume, RVMi RV indexed mass.
